# Promising survival rate but high incidence of treatment‐related mortality after reduced‐dose craniospinal radiotherapy and tandem high‐dose chemotherapy in patients with high‐risk medulloblastoma

**DOI:** 10.1002/cam4.3199

**Published:** 2020-06-30

**Authors:** Ji Won Lee, Do Hoon Lim, Ki Woong Sung, Hee Won Cho, Hee Young Ju, Ju Kyung Hyun, Keon Hee Yoo, Hong Hoe Koo, Yeon‐Lim Suh, Yoo‐Sook Joung, Hyung Jin Shin

**Affiliations:** ^1^ Department of Pediatrics Samsung Medical Center Sungkyunkwan University School of Medicine Seoul Republic of Korea; ^2^ Department of Radiation Oncology Samsung Medical Center Sungkyunkwan University School of Medicine Seoul Republic of Korea; ^3^ Department of Pathology Samsung Medical Center Sungkyunkwan University School of Medicine Seoul Republic of Korea; ^4^ Department of Psychiatry Samsung Medical Center Sungkyunkwan University School of Medicine Seoul Republic of Korea; ^5^ Department of Neurosurgery Samsung Medical Center Sungkyunkwan University School of Medicine Seoul Republic of Korea

**Keywords:** craniospinal radiotherapy, high‐dose chemotherapy, long‐term follow‐up, medulloblastoma, treatment‐related mortality

## Abstract

**Background:**

In this study, we report the follow‐up results of reduced dose of craniospinal radiotherapy (CSRT) followed by tandem high‐dose chemotherapy (HDCT) in patients with high‐risk medulloblastoma (MB).

**Methods:**

Newly diagnosed high‐risk MB patients (metastatic disease, postoperative residual tumor >1.5 cm^2^, or large cell/anaplastic histology) over 3 years of age were enrolled in this study. Two cycles of pre‐RT chemotherapy, radiotherapy (RT) including reduced‐dose CSRT (23.4 or 30.6 Gy), four cycles of post‐RT chemotherapy, and tandem HDCT were administered. NanoString and DNA sequencing were performed using archival tissues.

**Results:**

In all, 40 patients were enrolled, and molecular subgrouping was possible in 21 patients (2 wingless, 3 sonic hedgehog, 8 Group 3, and 8 group 4). All patients including two patients who experienced progression during the induction chemotherapy underwent HDCT. Relapse/progression occurred only in four patients (5‐year cumulative incidence [CI] 10.4 ± 0.3%). However, six patients died from treatment‐related mortality (TRM) (four acute TRMs and two late TRMs) resulting in 18.5 ± 0.5% of 5‐year CI. Taken together, the 5‐year event‐free survival and overall survival were 71.1 ± 8.0% and 73.2 ± 7.9%, respectively. Late effects were evaluated in 25 patients and high‐tone hearing loss, endocrine dysfunction, dyslipidemia, and growth retardation were common.

**Conclusions:**

The strategy using tandem HDCT following reduced‐dose CSRT showed promising results in terms of low relapse/progression rate; however, the high TRM rate indicates that modification of HDCT regimen and careful selection of patients who can benefit from HDCT will be needed in the future study.

## Graphical abstract

Tandem high‐dose chemotherapy (HDCT) after reduced‐dose craniospinal radiotherapy resulted in a low progression rate in high‐risk medulloblastoma. However, the high treatment‐related mortality rate indicates the need to modify the HDCT regimen and carefully select patients who may benefit from HDCT.

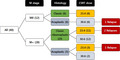

## 1 INTRODUCTION

Medulloblastoma (MB) is the most common malignant brain tumor of childhood accounting for 20% of CNS tumors in the pediatric population.^1^ Standard treatment of MB consisted of maximal surgical resection followed by risk‐adaptive craniospinal radiotherapy (CSRT) and adjuvant chemotherapy. The current clinical risk stratification divides MB into standard risk and high risk according to age, presence of metastasis, extent of postsurgical residual disease, and histology.^2^ In patients over 3 years old, patients who had subtotal resection, metastatic disease, and/or anaplastic histology are considered as high risk. Current treatment protocols for high‐risk MB generally use 36.0‐39.6 Gy CSRT followed by a tumor bed boost to 54.0‐55.8 Gy of total dose.^2^, ^3^ With this approach, the 5‐year event‐free survival (EFS) in high‐risk MB has been reported as 60%‐70%.^4^, ^5^

However, radiotherapy (RT), especially CSRT, can cause several serious late sequelae such as neurocognitive dysfunction, endocrine dysfunction, growth disturbances, and secondary malignancy.^6^ High‐dose chemotherapy (HDCT) with autologous stem cell rescue has been investigated in infant brain tumors including MB to reduce, defer, or omit RT to minimize the risk of irreversible sequelae of RT, particularly CSRT, and this approach showed success in some studies.^7^, ^8^ Also, some studies have suggested that further dose‐escalation using tandem HDCT might further improve outcomes in the treatment of recurrent or high‐risk brain tumors.^9^, ^10^ With this background, we hypothesized that the dose of CSRT might be reduced without jeopardizing survival rate if chemotherapy is intensified with tandem HDCT in high‐risk MB.

From 2005, we performed reduced‐dose CSRT followed by tandem HDCT in patients with high‐risk MB, and reported early results of this study in 2012 with 20 patients.^11^ However, the previous report had its limitations in terms of small number of patients, short follow‐up duration, and lack of molecular study. Therefore, we reported the results of longer follow‐ups with a larger number of enrolled patients. Molecular studies were also performed to investigate the effect of molecular subgroups on the clinical outcome in patients using this treatment strategy.

## 2 METHODS

### 2.1 Patients

Newly diagnosed high‐risk MB patients over 3 years of age were enrolled in this study from October 2005 to April 2018. High‐risk MB was defined as MB with metastatic disease (M+), postoperative residual tumor > 1.5 cm^2^ (R+), or large cell/anaplastic histology. This study was approved by the Institutional Review Board of our center, and written informed consent was obtained from the parents or guardians of each patient.

### 2.2 Treatment

Detailed information of treatment was described in the previous report.^11^ Briefly, two cycles of pre‐RT chemotherapy were given before RT, and four cycles post‐RT chemotherapy were given with reduced dose by 25% before tandem HDCT. A cisplatin‐etoposide‐cyclophosphamide‐vincristine regimen and a carboplatin‐etoposide‐ifosfamide‐vincristine regimen were used in alternation. Peripheral blood stem cells (PBSCs) were collected during the recovery phase after the first chemotherapy cycle.

Radiation dose was initially 23.4 Gy of CSRT, 30.6 Gy of primary site RT, and 21.6 Gy of boost to gross seeding nodule for all patients. After experiencing three relapses from nine M+ patients during the early study period (from October 2005 through December 2007), CSRT dose was increased to 30.6 Gy for M+ patients over 6 years of age or for patients with anaplastic MB. RT was administered at 1.8 Gy/d for 5 d/wk. All patients were treated with three‐dimensional photon beams until December 2015; thereafter, patients were treated with proton beams.

CTE (carboplatin, thiotepa, and etoposide) and CyM (cyclophosphamide and melphalan) regimens were used for the first and second HDCT, respectively. In 2014, we published a paper studying the toxicity during tandem HDCT using CTE/CyM regimen in brain tumor. In this paper, age younger than 8 years was a significant predictor of hepatic veno‐occlusive disease (VOD), and all six patients who died from treatment‐related mortality (TRM) during the second HDCT were younger than 9 years of age.^12^ With these results, we reduced the dose of tandem HDCT for young children: 90% for children aged ≥6‐9 years and 80% for ≥3‐6 years. As a result, 3 out of 6 patients aged ≥3‐6 years received 80% dose and 7 out of 12 patients aged ≥6‐9 years received 90% dose. Patients received heparin at a dose of 100 IU/kg/d and lipo‐prostaglandin E1 (Alprostadil, Eglandin; Mitsubishi Tanabe Pharma Co.) at a dose of 1 mg/kg/d through continuous infusion for prophylaxis of VOD. We allowed an approximate 12‐week interval without treatment between the first and second HDCT.

### 2.3 Surveillance of late complications

Late effects were evaluated annually after the second HDCT. Endocrine, ophthalmologic, auditory, cardiac, and respiratory problems were evaluated along with cognitive function. The diagnosis of growth hormone deficiency was based on a declining growth rate, and it was confirmed by biochemical testing. Hypothyroidism was diagnosed by elevated thyrotropin levels. Adrenal insufficiency was diagnosed on the basis of the failure to increase cortisol levels after corticotropin‐releasing hormone administration.

Cognitive function was evaluated using the Korean version of the Wechsler Intelligence Scales of Children (K‐WISC) and Korean version of Wechsler Adult Intelligence Scale (K‐WAIS). Korean version of the Wechsler Intelligence Scales of Children‐III and K‐WAIS‐III were used until 2011, and thereafter, K‐WISC‐IV and K‐WAIS‐IV were used. Korean version of the Wechsler Intelligence Scales of Children‐III/K‐WAIS‐III used 10 subtests to calculate the Verbal intelligence quotient (IQ) Scale and Performance IQ scale score; 5 subtests (Information, Similarities, Arithmetic, Vocabulary, and Comprehension in K‐WISC‐III/Information, Similarities, Arithmetic, Vocabulary, and Digit span in K‐WAIS‐III) for Verbal IQ and 5 (Picture Completion, Coding, Picture Arrangement, Block Design, and Object Assembly in K‐WISC‐III and Block Design, Matrix Reasoning, Visual Puzzles, Symbol Search, and Coding in K‐WAIS‐III) for Performance IQ. Full‐Scale IQ (FSIQ) is a composite of the Verbal and Performance scores. In the K‐WISC‐IV and K‐WAIS‐IV, 10 core subtests are used to create 4‐factor index scores, and FSIQ is computed from all 10 core subtests.

### 2.4 Molecular study

Formalin‐fixed, paraffin‐embedded tissues were used, and all tumor specimens were reviewed by a pathologist to determine the percentage of viable tumor and their adequacy for molecular tests. For molecular subgrouping, the nCounter^®^ system (NanoString Technologies) was used according to the methods proposed by Northcott et al in 2012.^13^ All procedures were performed according to the manufacturer's instructions. A customized cancer panel, designed to cover the exonic DNA sequences of 351 cancer genes was used for DNA sequencing. DNA preparation, panel sequencing, and bioinformatics analysis were performed as previously published.^14^

### 2.5 Statistics

Differences in continuous variables were calculated using Mann‐Whitney *U* test. EFS rate and overall survival (OS) rate were estimated using the Kaplan‐Meier method, and the difference of the survival curve was compared with the log‐rank test. Cumulative incidences (CI) of relapse/progression and TRM were estimated with competing risk methods.^15^ Relapse/progression and TRM were regarded as competing risks with each other.

## 3 RESULTS

### 3.1 Patients characteristics

From October 2005 to April 2018, 40 patients (23 males and 17 females) were enrolled in this study. The median age at diagnosis was 8.5 years (range, 3.8‐31.5 years). Nineteen (47.5%) patients had gross residual tumor > 1.5 cm^2^ after surgery, and 28 (70.0%) patients had metastatic disease at initial diagnosis. M stages were M1 in 1 patient, M2 in 8, M3 in 18 patients, and M4 in 1 patient. Histologically, 26 patients had classic types, 3 had desmoplastic/nodular types, and 11 had anaplastic types.

### 3.2 Molecular subgroup

**FIGURE 1:**
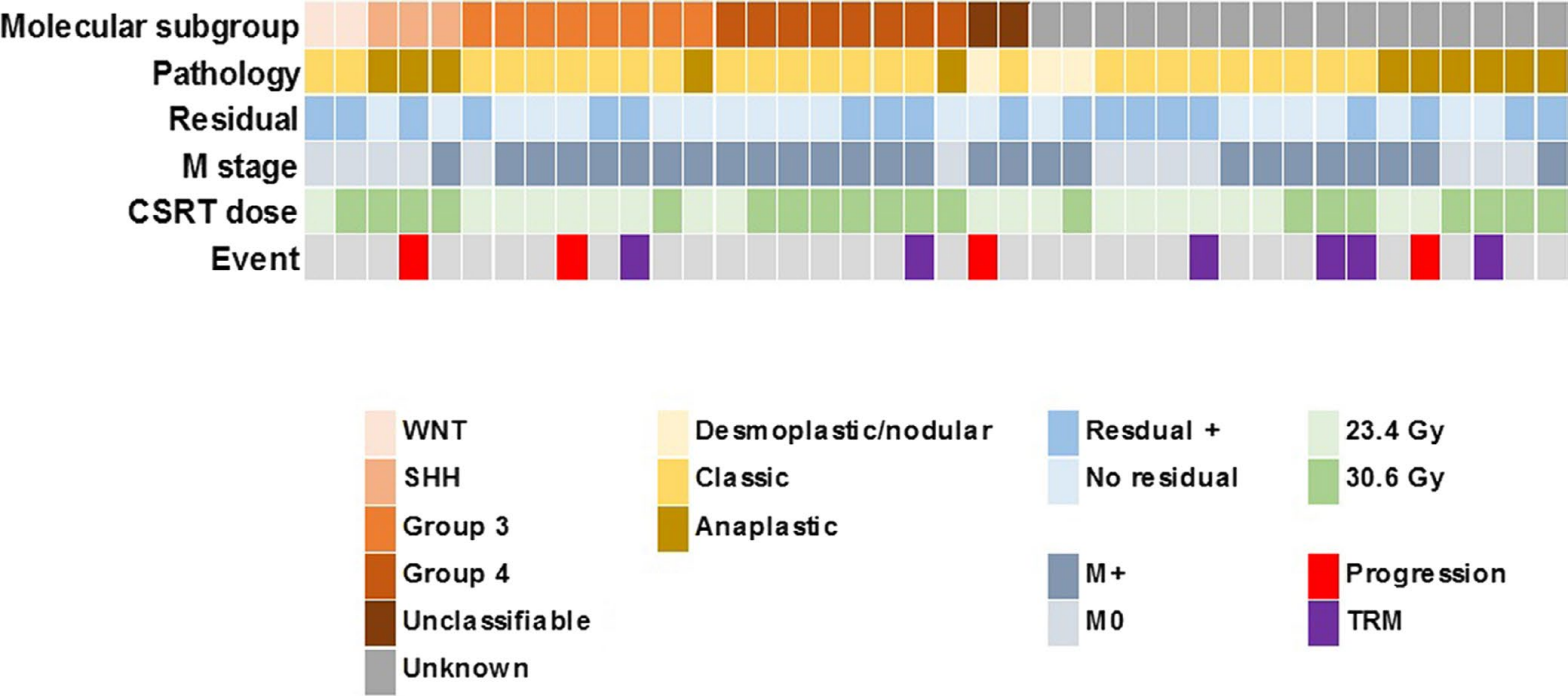
Molecular subgroups of patients. Molecular subgroups incorporating the results of NanoString and DNA sequencing are illustrated along with the clinical features and outcomes. CSRT, craniospinal radiotherapy; TRM, treatment‐related mortality

Among the 40 patients, tissues at diagnosis were available for 25 patients. The results of NanoString assay and Next‐generation Sequencing (NGS) study are summarized in Table 1. After RNA extraction, three RNA samples were excluded because they did not meet the quality standard. NanoString assay of samples of the remaining 22 patients successfully classified 19 patients into 1 wingless (WNT), 2 sonic hedgehog (SHH), 8 Group 3, and 8 Group 4. The remaining three patients were not classifiable with the NanoString method because the results after class prediction analysis did not fit any subtype. Panel sequencing of DNA could be performed in 17 patients. It revealed *CTNNB1* mutations (1 S33P and 1 S33F) with loss of chromosome 6 in two patients (one WNT and one unclassifiable in NanoString assay). One patient who was not classifiable with NanoString method had stopgain (S733*, VAF 37.27%) and frameshift (L431fs, VAF 4.14%) mutation of *PTCH1*. *TP53* mutation (R158H) was found in only one WNT patient. No *MYCN* amplification was observed in these patients. Taking the NanoString assay and panel sequencing results together, we could classify 21 patients into subgroup; 2 WNT, 3 SHH, 8 Group 3, and 8 group 4. Figure 1 illustrates the molecular subgroup along with their clinical and pathological features.

**TABLE 1:** Results of molecular study

Patients no	NanoString assay	Panel sequencing^a^	Subgroup
2	Group 3	N‐S	Group 3
4	Fail	Fail	fail
5	Unclassifiable	Fail	Unclassifiable
7	Fail	Fail	fail
8	Group 3	Fail	Group 3
10	Group 4	N‐S	Group 4
11	SHH	Fail	SHH
12	WNT	*CTNNB1* S33F, Chr 6 loss, *TP53* R158H	WNT
13	Group 4	Fail	Group 4
14	Group 4	Fail	Group 4
16	Group 3	*BRCA2* 1858_1859 del	Group 3
19	Unclassifiable	N‐S	Unclassifiable
21	Group 4	N‐S	Group 4
22	SHH	Fail	SHH
23	Group 4	N‐S	Group 4
25	Fail	*CTNNB1* S33P, Chr 6 loss	WNT
27	Group 3	N‐S	Group 3
28	Group 3	N‐S	Group 3
29	Group 4	N‐S	Group 4
30	Group 4	N‐S	Group 4
32	Group 4	N‐S	Group 4
34	Group 3	*TSC1* G389*, *CDKN2A/2B* del	Group 3
37	Unclassifiable	*PTCH1* S733*, *PTCH1* L431fs, *PIK3CA* E542K	SHH
39	Group 3	N‐S	Group 3
40	Group 3	*SMARCA4* T910M	Group 3

Abbreviations: del, deletion; fs, frameshift mutation; N‐S, no significant alteration; SHH, sonic hedgehog; WNT, wingless.

^a^ Only pathogenic or likely pathogenic variants were illustrated.

### 3.3 Treatment of patients

**FIGURE 2:**
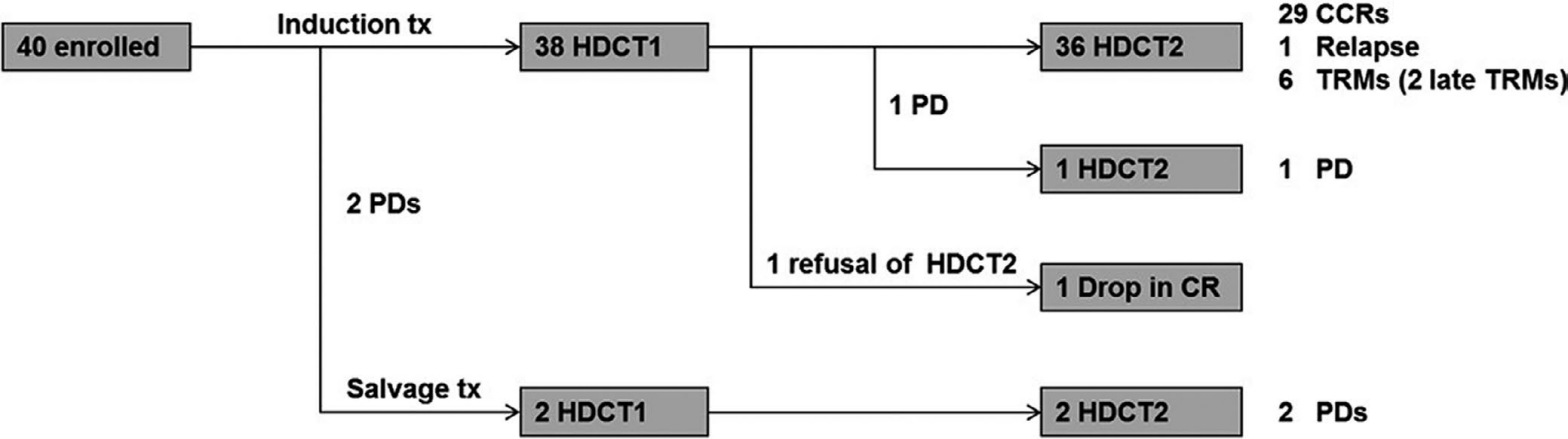
Flow of patients. Treatment courses of enrolled patients are summarized. All enrolled patients including two patients who showed progression during the induction chemotherapy underwent the first HDCT. Second HDCT was given to all patients except 1 patient who refused to proceed to second HDCT. CCR, continuous complete remission; HDCT, high‐dose chemotherapy; PD, progressive disease; TRM, treatment‐related mortality

Figure 2 shows the flow of enrolled patients. All enrolled patients including two patients who showed progression during the post‐RT chemotherapy underwent the first HDCT. The two patients who experienced progression during post‐RT chemotherapy received additional RT and salvage chemotherapy with topotecan/cyclophosphamide. One patient showed a partial response after salvage treatment, but the other patient showed progression. Both of them proceeded to tandem HDCT. Second HDCT was administered to all patients except one patient who refused to proceed to second HDCT. One patient who experienced relapse after the first HDCT also proceeded to the second HDCT as salvage treatment.

**FIGURE 3:**
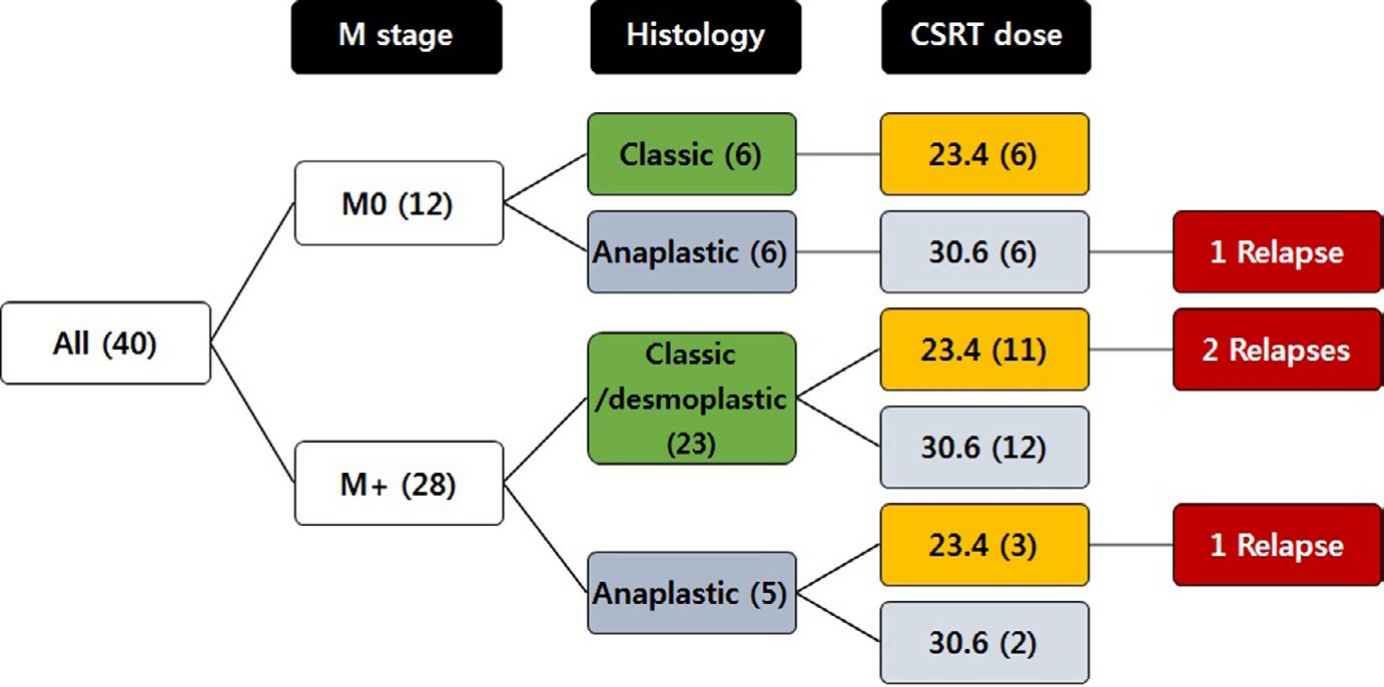
Relapse according to M stage, histology, and CSRT dose. Treatment outcome according to the M stage, histology, and CSRT dose is illustrated. The numbers in parentheses indicate the number of patients in each group. CSRT, craniospinal radiotherapy

Craniospinal radiotherapy dose was 23.4 Gy in 20 patients (6 M0, 14 M+) and 30.6 Gy in 20 patients (6 M0, 14 M+). Craniospinal radiotherapy dose according to the M stage and histology is illustrated in Figure 3. In nine patients, boost RT was administered to a gross metastatic nodule. Proton beam therapy was administered to 11 patients.

During the tandem HDCT, the median numbers of infused CD34^+^ cells were 35.4 (10.9‐98.0) × 10^6^/kg in the first HDCT and 35.6 (7.6‐157.0) × 10^6^/kg in the second HDCT. The median number of days required to reach an Absolute Neutrophil Count of more than 0.5 × 10^9^/L was 8 days (7‐10 days) in the first HDCT and 9 days (7‐12 days) in the second HDCT. Platelet recovery more than 20 × 10^9^/L required a median 19 days (13‐99 days) in the first HDCT and 22.5 days (13‐294 days) in the second HDCT. Among 15 patients who underwent HDCT after 2015, the dose of HDCT was reduced in 10 patients under 9 years of age; 90% in 7 patients and 80% in 3 patients. Acute toxicities ≥ Common Terminology Criteria for Adverse Events grade 3 during the tandem HDCT are summarized according to the study period in Table 2. The frequencies of stomatitis, diarrhea, liver enzyme elevation, and hypokalemia were higher in the first HDCT. Hepatic VOD occurred in nine patients (23.1%) during the second HDCT; four of them recovered with conservative management, and the other five received intensive care management including continuous renal replacement therapy and ventilator care.

**TABLE 2:** Acute toxicities after HDCT

Parameters	HDCT1 (n = 40)		HDCT2 (n = 39)	
	Until 2014 (n = 25)	After 2015 (n = 15)	Until 2014 (n = 25)	After 2015 (n = 14)
Hematologic toxicity				
CD34^+^ cells (×10^6^/kg)^a^	42.5 (11.1‐98.0)	28.1 (10.9‐57.5)	37.1 (9.5‐157.0)	33.1 (7.6‐97.3)
Days to reach an ANC 500/μL^a^, ^b^	8 (7‐10)	8 (7‐10)	9 (8‐12)	8 (7‐9)
Days to reach a PLT count 20 000/μL^a^, ^c^	18 (13‐35)	21 (14‐99)	22 (13‐294)	24 (15‐88)
Days of BT ≥ 38.0°C, d (range)^a^	5 (0‐10)	2 (0‐6)	2 (0‐7)	0 (0‐3)
Positive blood culture, no. (%)	2 (8.0)	2 (13.3)	4 (16.0)	2 (14.3)
Non‐hematologic toxicity				
Stomatitis, no. (%)	21 (84.0)*	7 (46.7)*	2 (8.0)	0 (0.0)
Vomiting, no. (%)	4 (16.0)	1 (6.7)	2 (8.0)	2 (14.3)
Diarrhea, no. (%)	10 (40.0)	3 (20.0)	6 (24.0)	5 (35.7)
Elevation of liver enzyme, no. (%)	15 (60.0)	9 (60.0)	1 (4.0)	0 (0.0)
Hyperbilirubinemia, no. (%)	1 (4.0)	1 (6.7)	2 (8.0)	0 (0.0)
Renal insufficiency, no. (%)	0 (0.0)	0 (0.0)	2 (8.0)	0 (0.0)
Hypokalemia, no. (%)	13 (52.0)	4 (26.7)	10 (40.0)*	0 (0.0)*
Hyperkalemia, no. (%)	2 (8.0)	1 (6.7)	2 (8.0)	0 (0.0)
Hyponatremia, no. (%)	0 (0.0)	0 (0.0)	2 (8.0)	1 (7.1)
Hypernatremia, no. (%)	1 (4.0)	0 (0.0)	0 (0.0)	0 (0.0)
Hepatic VOD, no. (%)	0 (0.0)	1 (6.7)	7 (28.0)	2 (14.3)
Myocarditis, no. (%)	0 (0.0)	0 (0.0)	0 (0.0)	0 (0.0)

Abbreviations: ANC, absolute neutrophil count; BT, body temperature; HDCT, high‐dose chemotherapy; PLT, platelet; VOD, veno‐occlusive disease.

^a^ Median (range).

^b^ The first day ANC exceeded 500 neutrophils/mL for 3 consecutive days.

^c^ The first day PLT count exceeded 20 000 platelets/mL without transfusion for 7 d.

* *P* < .05

### 3.4 Events and survival

Treatment‐related mortality occurred in six patients (four acute TRMs and two late TRMs) resulting in 18.5 ± 0.5% of 5‐year CI. Four patients died from acute TRM during the second HDCT. Three of them died from hepatic VOD and multi‐organ failure (MOF) at 1.9, 2.3, and 3.3 months after the second HDCT. All of them were under 9 years at the time of second HDCT, and two of them underwent HDCT after 2016 and received reduced dose (90%) of HDCT. Another patient with acute TRM died of RSV pneumonia 1.1 months after the second HDCT. Two patients died from late TRMs. One of them had severe VOD and acute renal failure during the second HDCT, which progressed to chronic renal failure. The patient admitted to the hospital with Influenza A viral pneumonia and died of progressive interstitial lung disease and acute respiratory distress syndrome (ARDS) at 30 months after the HDCT. The other patient had been suffering from recurrent infections such as pneumonia, urinary tract infection, peritonitis, and ventro‐peritoneal shunt infection after the HDCT, and died from pneumonia/ARDS at 46 months after the second HDCT.

**FIGURE 4:**
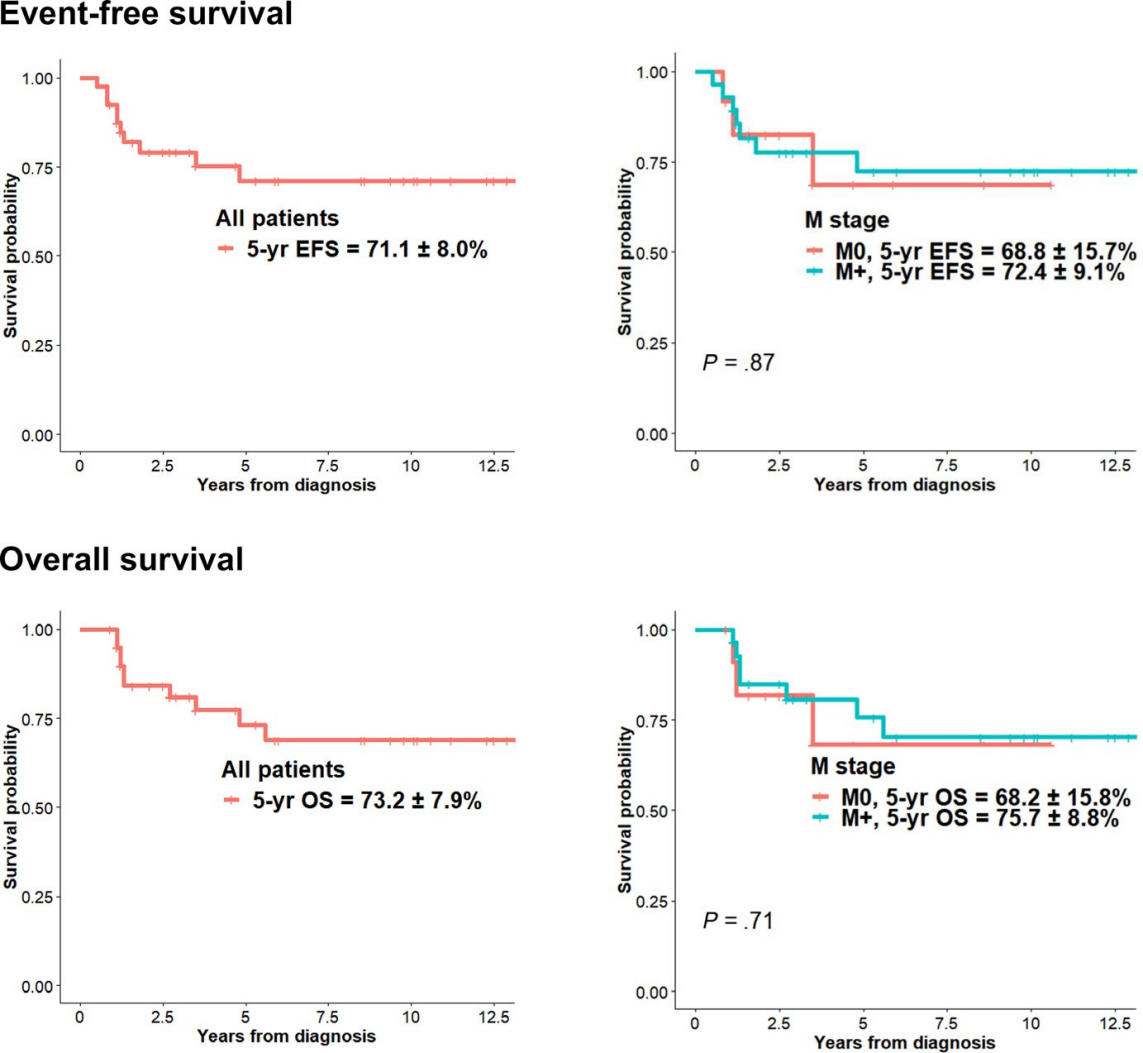
Survival of patients. Event‐free survival and overall survival of all patients and the differences according to the M stage are analyzed

Relapse/progression occurred in four patients (three at the spinal cord and one at the primary site) with 10.4 ± 0.3% of 5‐year CI; two during the induction chemotherapy, one after the first HDCT, and one after the second HDCT. After the change of RT strategy from 2008 (increased CSRT dose to 30.6 Gy for M+ patients over 6 years or for patients with anaplastic MB), only one patient with R+/M0 and anaplastic histology experienced relapse. Relapse/progression according to the M stage, histology, and CSRT dose is illustrated in Figure 3. Of 12 M0 patients, only one patient who had an anaplastic tumor and had a significant postoperative residual tumor experienced relapse at the primary site within the local RT field after receiving 30.6 Gy of CSRT/23.4 Gy of local RT. No recurrence was observed in six patients with M0/classic histology who received 23.4 Gy of CSRT. Among 14 M+ patients who received 23.4 Gy of CSRT, three experienced relapses and all of them were metastatic relapses at the spinal cord. All 14 M+ patients who received 30.6 Gy of CSRT remain progression free regardless of histology. According to the molecular subgroup, one patient with SHH MB and one patient with Group 3 MB had relapse/progression (Figure 1). All of the four patients who experienced relapse/progression eventually died despite salvage treatments, and as a result, the 5‐year EFS and OS of all patients were 71.1 ± 8.0% and 73.2 ± 7.9%, respectively. There were no differences in EFS and OS according to the M stage (Figure 4).

### 3.5 Late effects and long‐term outcome

**FIGURE 5:**
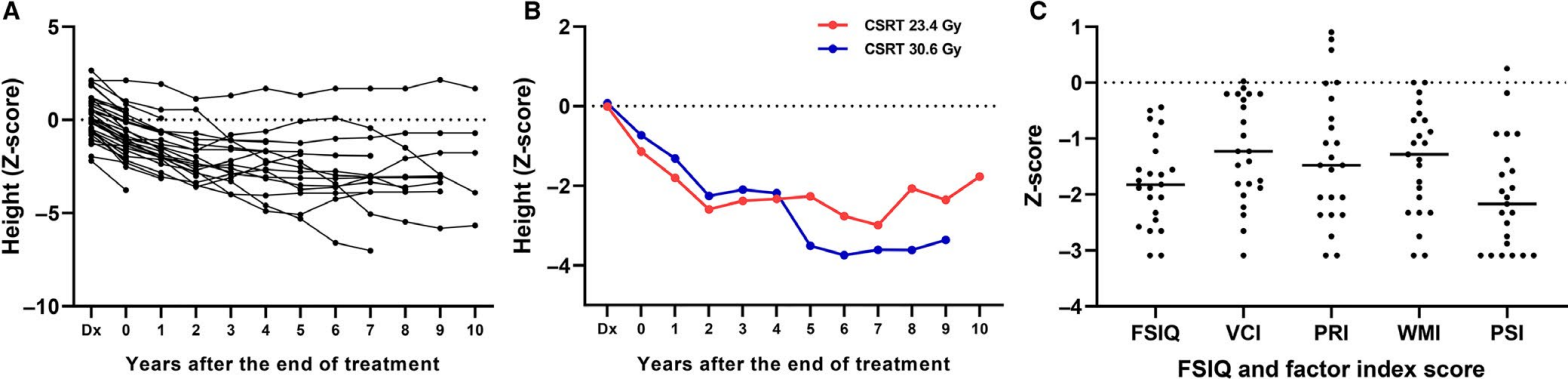
Late effects. A, Annual *Z*‐score of heights of each patient are plotted. B, Patients who received 23.4 Gy of CSRT seemed to have better results in the growth retardation, but there was no statistical significance. C, *Z*‐score of FSIQ and 4‐factor index score of K‐WISC‐IV or K‐WAIS‐IV are plotted. CSRT, craniospinal radiotherapy; FSIQ, Full‐Scale intelligence quotient; PRI, perceptual reasoning index; PSI, processing speed index; VCI, verbal comprehension index; WMI, working memory index

Late effects were evaluated in 25 patients including 6 patients who were treated with proton beams. Late effect evaluations were performed periodically, and the results of last evaluation for each patient were used for the analysis. Median age of patients at the evaluation was 19.2 years (range, 7.6‐36.5) years, and median time after the second HDCT was 7.5 years (range, 1.2‐12.3 years). Summary of late effects is illustrated in Table 3. High‐tone hearing loss was the most prevalent late effect, but only two patients needed hearing aids. Endocrine problems and dyslipidemia were also common. Five patients developed grade 1 chronic kidney disease (median estimated glomerular filtration rate 74.5 mL/min/1.73 m^2^, range 72.3‐84.0 mL/min/1.73 m^2^), and none of these patients experienced VOD during the tandem HDCT. One patient who received 23.4 Gy of CSRT developed meningioma on the anterior falx at 13 years after initial diagnosis. Vertical growth was evaluated excluding the five patients who were diagnosed after 18 years of age (Figure 5A). Median *Z*‐score at 5 years after the second HDCT was −2.66 (range, 5.31‐1.33). Patients who received 23.4 Gy of CSRT seemed to have better results in the growth retardation, but there was no statistical significance (Figure 5B).

**TABLE 3:** Late complications

Late complications	Until 2014 (n = 17)		After 2015 (n = 8)	
	No.	%	No.	%
Endocrine				
Hypothyroidism	9	52.9	1	12.5
Growth hormone deficiency	10	58.8	2	25.0
Glucocorticoid deficiency	2	11.8	0	0.0
Sex hormone deficiency^a^	7	41.2	2 (n=3)	66.7
Precocious puberty	1	5.9	1	12.5
Dyslipidemia	7 (2)^b^	41.2	1	12.5
Hearing loss	11 (2)^b^	64.7	7	87.5
Opthalmologic				
Cataract	2	11.8	0	0.0
Optic neuropathy	0	0.0	1	12.5
Chronic lung disease	0	0.0	0	0.0
Renal				
Chronic kidney disease	3	17.6	2	25.0
Cardiac	0	0.0	0	0.0
Heart dysfunction	0	0.0	0	0.0
Arrhythmia	1 (1)^b^	5.9	0	0.0
Others				
Meningioma	1	5.9	0	0.0
SMA syndrome	0	0.0	1	12.5
Barrett esophagus	1	5.9	0	0.0
NAFLD	1	5.9	0	0.0
Genu valgum	1	5.9	0	0.0
Spastic hand deformity	1	5.9	0	0.0

^a^ Sex hormone deficiency was evaluated in patients who reached puberty.

^b^ The numbers in parenthesis indicate the number of patients with CTCAE grade 3 or higher.

Cognitive function test was performed at median 4.8 (range, 0.9‐10.1) years after the second HDCT. Korean version of the Wechsler Intelligence Scales of Children‐III or K‐WAIS‐III were used in 2 patients, and K‐WISC‐IV or K‐WAIS‐IV were used in the remaining 23 patients. Median values for FSIQ were 71 (range, 47‐108); ≤69 in 8 patients, 70‐79 in 9 patients, and ≥80 in 8 patients. The detailed results of patients who had K‐WISC‐IV or K‐WAIS‐IV are illustrated in Table 4 and Figure 5C.

**TABLE 4:** Cognitive function test

K‐WISC‐IV (N = 11)	Mean	SD	Range	K‐WAIS‐IV (N = 12)	Mean	SD	Range
*Verbal comprehension index*				*Verbal comprehension index*			
Similarities	4.8	3.5	1‐11	Similarities	9.1	2.8	4‐13
Vocabulary	6.5	2.5	2‐10	Vocabulary	7.4	3.0	3‐12
Comprehension	5.4	3.0	1‐10	Information	8.6	2.6	5‐14
*Perceptual reasoning index*				*Perceptual reasoning index*			
Block design	7.1	4.3	1‐13	Block design	6.0	3.5	3‐13
Picture concepts	6.0	3.8	1‐11	Matrix reasoning	8.3	2.9	3‐12
Matrix reasoning	6.4	4.7	1‐13	Visual puzzle	6.9	2.3	3‐12
*Working memory index*				*Working memory index*			
Digit span	5.5	3.0	1‐9	Digit span	6.7	4.0	1‐12
Letter‐number sequencing	6.5	2.7	2‐10	Arithmetic	7.3	2.2	2‐10
*Processing speed index*				*Processing speed index*			
Coding	3.2	2.0	1‐8	Symbol search	4.7	2.5	1‐9
Symbol search	4.5	3.7	1‐11	Coding	5.5	3.7	1‐12

Abbreviations: K‐WAIS, Korean version of Wechsler Adult Intelligence Scale; K‐WISC, Korean version of Wechsler Intelligence Scales of Children; SD, standard deviation.

There was no significant difference in the long‐term outcome including endocrinologic problems, height, and cognitive function according to the RT modalities. With respect to educational attainment and employment, among the 14 patients currently over 18 years of age (two patients were diagnosed at adult age), 10 entered university after the end of treatment. Among five patients over 23 years of age, three had jobs.

## 4 DISCUSSION

In this study, tandem HDCT was performed to reduce the CSRT dose to 23.4 or 30.6 Gy in high‐risk MB patients. This approach resulted in promising survival rate showing 10.4% of CI of relapse/progression and 71.1% of 5‐year EFS, and the EFS was not different according to the presence of metastasis. EFS of high‐risk MB remained less than 50% in the earlier studies until 1990.^16^, ^17^, ^18^ Many different strategies, including HDCT, hyperfractionated accelerated radiotherapy, or concurrent chemotherapy during CSRT, have been employed to overcome the poor outcome in the last decades.^4^, ^10^, ^19^, ^20^ In the HIT 2000 trial with metastatic MB incorporating 40.0 Gy of hyperfractionated CSRT showed 5‐year EFS and OS were 62% and 74%, respectively.^4^ Children's Oncology Group (COG) studied concurrent carboplatin during 36 Gy of CSRT as a radiosensitizer in metastatic MB, and 5‐year EFS and OS were reported as 78% and 71%, respectively.^20^ In a study by Gajjar et al, 48 patients with high‐risk MB were treated with CSRT (36‐39.6 Gy) followed by four cycles of HDCT and autologous stem cell rescue, and the 5‐year EFS rate was 70%.^10^ Considering that the CSRT dose of the previous studies was 36‐40 Gy, it is meaningful that our study showed similar EFS using 23.4‐30.6 Gy of CSRT. These findings suggest that, in patients with high‐risk MB, dose‐intense chemotherapy may reduce the necessary dose of CSRT. In our study, all of the six patients with M0/classic histology (who were classified into high‐risk MB due to the presence of residual tumor) were progression free with 23.4 Gy of CSRT. Also, all 14 M+ patients who received 30.6 Gy of CSRT remain progression free. These results can suggest that CSRT dose could be reduced to 30.6 Gy if tandem HDCT is combined even in M+ patients.

However, the biggest problem of the study was the high incidence of TRM. Especially, hepatic VOD was the main cause of TRM. During the second HDCT, hepatic VOD occurred in nine (23.1%) patients, and three of them died of progressive hepatic VOD and MOF. Tandem HDCT is associated with greater toxicity and a higher TRM rate than single HDCT, particularly during the second HDCT. The variable intensity of tandem HDCT regimens could result in different outcomes and toxicity profiles.^10^, ^19^ A more intensive tandem HDCT regimen is associated with a higher TRM rate but also might be associated with a lower relapse/progression rate. The optimal combination of regimens for tandem HDCT has not yet been determined. In our institution, the CTE/CyM regimen has been used since 2005 after these two regimens have separately shown efficacy in pediatric high‐risk or recurrent brain tumors.^21^, ^22^ In our previous study reporting the toxicity during tandem HDCT using CTE/CyM regimen, age younger than 8 years was the significant predictor for hepatic VOD, and all six patients who died from toxicity during the second HDCT were younger than 9 years of age.^12^ Due to these results, we reduced the dose of HDCT according to the age at HDCT during the late study period. Despite the dose reduction strategy, two young patients still developed hepatic VOD and died. This high rate of TRM cannot be accepted even considering the severity of the patient's disease. Therefore, further study is needed to determine the optimal dose of HDCT especially in young children. In this study, infused stem cell doses were higher than the usual dose because stem cells were collected during the first cycle of chemotherapy. However, it is unlikely that the higher stem cell dose had an effect on the high TRM considering the stem cell dose of patients with TRM.

In this study, three patients showed disease progression while on treatment (two during the post‐RT chemotherapy and one before the second HDCT). All of them eventually died of disease progression even after the tandem HDCT. A patient who achieved PR after salvage treatment and underwent tandem HDCT remained progression free for 18 months after the second HDCT, but the other two patients died shortly after the second HDCT. Given the high toxicity of HDCT and the results of such treatment, HDCT should be carefully considered in patients who progressed during treatment, especially in patients who did not respond to the salvage treatment.

Gene expression profiling in MB by several study groups identified discrete molecular subgroups within MB, and consensus for MB subgroups was established in 2012 proposing four subgroup designations: WNT, SHH, Group 3, and Group 4.^23^ It is known that the four molecular subgroups differ in many aspects such as genetics, demographics, clinical features, and prognosis.^24^ Risk stratification of non‐infant, childhood MB (age: 3‐17 years) in the context of subgroups was further refined at the consensus conference in 2015,^25^ and the most recent clinical trials in MB have incorporated molecular risk stratification into the trial design.^3^ In the present study, we retrospectively performed NanoString and DNA sequencing with archival tissues to investigate the effect of molecular subgroups on the clinical outcome of this study. NanoString assay successfully classified 19 patients into subgroups, and two additional patients could be classified into WNT and SHH subgroups, respectively, because of the pathogenic mutation of pathognomonic genes of each subgroup such as *CTNNB1* or *PTCH1*. There were insufficient number of patients in each subgroup, and a few patients had relapses; thus, the number of patients was insufficient to tell the difference according to the molecular subgroups. Two patients with WNT MB were allocated in the high‐risk group due to the presence of residual tumor. Considering the good prognosis with standard treatment in WNT MB, this group of patients needs to be excluded in our future trial. Also, real‐time molecular study and prospective risk stratification will be needed for the future clinical trial.

In the late effect evaluation, endocrine dysfunction and high‐tone hearing loss were frequent, but grade 3‐4 toxicity was not common. There was no patient who developed secondary neoplasm except one patient who had meningioma. Cognitive function after pediatric brain tumor treatment has been one of the main concerns. It has been known that adult pediatric brain tumor survivors exhibited progressive decline in IQ scores because of the slow rate of acquisition of new information and skills.^26^ A conceptual model suggested that core cognitive abilities (such as working memory, information processing speed, and attention) underlie poor intellectual outcomes and academic achievements in pediatric medulloblastoma survivors,^26^, ^27^ and this theoretical model was empirically evaluated in a recent study.^28^ The cognitive function in our study showed similar results in terms of IQ score compared to the previous studies despite reducing the CSRT dose,^29^, ^30^ and this can be partly because HDCT can also negatively affect the cognitive outcome.^30^ In particular, among the four indices of the cognitive function test, the z‐score of the processing speed index (PSI) was lower than the other 3 index scores, in our study. Considering the results of King et al^28^ that processing speed was the core cognitive skill most widely associated with neurodevelopmental risk factors, future studies will need to carefully evaluate changes in the PSI to predict prognosis of cognitive function. For the potential future direction, germline genomics of the host could be one of the predictive markers of individual variation in neurocognitive morbidity after the treatment of pediatric brain tumor.^31^ In this study, there were no significant differences in late effects according to the RT modalities, but a longer follow‐up duration is needed in patients who were treated with proton beams.

In the present study, HDCT was effective in reducing progression rate while using lower CSRT dose, but it also showed severe toxicities. In the next phase, it is necessary to balance the efficacy and toxicity of HDCT. Reducing the intensity of HDCT may contribute to decrease not only the TRM but also the late effects. However, there are also concerns about how this strategy will affect survival. Another strategy that can be used is to consider the introduction of intrathecal (IT) methotrexate. Intraventricular or intravenous methotrexate has been used in some protocols showing promising results.^32^, ^33^ In the German HIT 2000 trial of non‐metastatic MB patients younger than 4 years, intraventricular and high‐dose intravenous methotrexate were combined with conventional chemotherapy resulting in 80 ± 6% of 5‐year OS.^32^ HIT 2000 trial including children and young adolescents also showed that a higher cumulative dose of intraventricular methotrexate was associated with better survival although there were frequent infectious complications associated with reservoir.^33^ Because there is still a problem with late sequelae even after the reduced‐dose RT, introduction of IT methotrexate could be considered as an alternative option to further reduce the RT dose.

In conclusion, strategy using tandem HDCT following reduced‐dose CSRT was successful in terms of low progression rate. However, the high TRM rate indicates that modification of HDCT regimen and careful selection of patients who can benefit from HDCT will be needed in future studies.

## ETHIC STATEMENT

This study was approved by the Institutional Review Board of our center (IRB No. 2005‐12‐009 and No. 2015‐11‐053) and written informed consent was obtained from the parents or guardians of each patient.

## CONFLICT OF INTEREST

The authors declare that they have no competing interests.

## AUTHORS' CONTRIBUTION

Study design: KWS, DHL, and HJS; clinical data collection and analysis: JWL, KWS, HWC, HYJ, JKH, KHY, HHK, and YSJ; genomic data collection and analysis: JWL and Y.S; article writing: JWL, KWS, and DHL; review the data and article: KWS, DHL, YS, and HJS.

## Data Availability

All data used in the production of this manuscript are available on request.
